# Assessing the readability and quality of online information on anosmia

**DOI:** 10.1308/rcsann.2022.0147

**Published:** 2023-04-13

**Authors:** H Raja, S Lodhi

**Affiliations:** ^1^ University Hospitals Birmingham NHS Foundation Trust, UK; ^2^ Manchester University NHS Foundation Trust, UK

**Keywords:** Anosmia, Health information, Readability, Coronavirus, Smell, Rhinology

## Abstract

**Introduction:**

Anosmia can have a significant impact on well-being and quality of life. Due to an ageing population and the coronavirus disease 2019, increasing numbers of patients are seeking online information on anosmia. This report systematically assesses the readability and quality of online information on anosmia.

**Methods:**

The terms ‘anosmia’ and ‘loss of smell’ were entered into Google. The first 50 websites generated for each search term were screened. Readability was assessed using the Flesch–Kincaid Reading Ease Score (FRES), Flesch–Kincaid Grade Level (FKGL), Simple Measure of Gobbledygook (SMOG) Index and Gunning Fog Index (GFI). Quality was assessed using the DISCERN instrument. Spearman’s correlation between quality and readability was calculated.

**Results:**

A total of 79 websites met the inclusion criteria. The mean and 95% confidence interval for the FRES, FKGL, SMOG, GFI and DISCERN scores were 46.31 (42.94–49.68), 12.00 (11.27–12.73), 10.70 (10.16–11.23), 14.62 (13.85–15.39) and 2.90 (2.69–3.11), respectively. Significant negative correlation was noted between the DISCERN and FRES (*r*_s_=−0.500; *p*<0.05).

**Discussion:**

Online information on anosmia is written above the recommended reading age guidance in the UK, and has moderate deficiencies in quality. As a result, the information may be used inappropriately and could result in worse health outcomes. We recommend that patients are directed to websites produced by health providers or nonprofit organisations that develop material for patient health education.

**Conclusions:**

Online information on anosmia is of low readability and moderate quality. Healthcare professionals should direct patients towards high-quality resources written for the layperson.

## Introduction

Anosmia, the loss of olfactory function, affects approximately 5–20% of the UK population.^1^ It is a symptom that has gained considerable media and scientific attention following the coronavirus disease 2019 (COVID-19) pandemic, an infectious disease caused by the severe acute respiratory syndrome coronavirus 2 (SARS-CoV-2). Pooled global prevalence of anosmia in COVID-19 cases has been reported to be approximately 40%, with around one in four individuals reporting the loss of their sense of smell as the first symptom experienced from the disease process.^2,3^

Studies have demonstrated that profound psychosocial effects secondary to anosmia, such as depression and concerns regarding personal hygiene, can reduce quality of life.^4^ Individuals with anosmia can also feel vulnerable to potential hazards from environmental dangers such as gas leaks and spoiled food. Accessing timely medical information to minimise feelings of mental, physical and social vulnerability can, therefore, be a key priority for patients suffering from anosmia.

Since the beginning of the COVID-19 pandemic, there has been a considerable transformation in UK healthcare delivery. To reduce the transmission of SARS-CoV-2 and alleviate the strain on the NHS, there has been an increased uptake of telehealth, in both primary and secondary care.^5-7^ Similarly, the pandemic has changed the dynamic of health-seeking behaviour of patients, with greater reliance on performing health-related online searches.^8^ Due to a lack of regulation, however, online websites can be of variable readability and quality. Patients with anosmia can ill afford this as it can have a negative impact on health-related choices and outcomes.^9,10^

To date, no study has examined the appropriateness of online information on anosmia. This study aimed to assess the readability and quality of online information on anosmia.

## Methods

**Figure 1:**
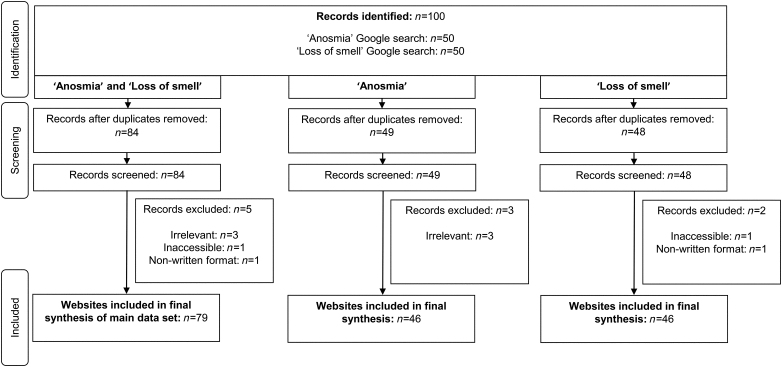
Flow diagram displaying the systematic search methodology. The searches were performed in November 2021.

The search terms ‘anosmia’ and ‘loss of smell’ were entered into the Google search engine in November 2021. Before performing the search, cookies and browser history were deleted to prevent the search results from being altered by previous internet usage. Google was used as the search engine of choice due to it occupying approximately 92% of the overall market share worldwide.^11^ Research has also shown that 90% of search engine users click on a link in the first three search result pages (30 results).^12^ Therefore, to ensure a thorough search was conducted, the first 50 results for each search were screened. The workflow of our methodology is shown in Figure 1.

### Eligibility criteria

Only websites in the English language were included. Websites that were duplicates, inaccessible, presented in a nonwritten format, or composed of only irrelevant information were excluded from the analysis.

Two independent assessors (HR and SL) screened the websites to determine their eligibility for inclusion, and calculated readability and quality scores. A third independent assessor was available to make a final decision if any interassessor inconsistencies arose.

### Readability assessment

Four validated readability tests were used to complete objective assessment of readability: Flesch Reading Ease Score (FRES), Flesch–Kincaid Grade Level (FKGL), Simple Measure Of Gobbledygook (SMOG) Index and Gunning Fog Index (GFI). The FRES is calculated using the following formula: 206.835–1.015×(total number of words ÷ total number of sentences) −84.6×(total number of syllables ÷ total number of words). This generates a score of 0–100 for each source, with a higher score corresponding to the content being easier to read (e.g., 0–30: very difficult; 90–100: very easy).^13^

The FKGL, SMOG Index and GFI generate a score between 0–12, 4–18 and 0–20, respectively. The scores are estimates of the US grade level of education required to comprehend the information (e.g., 6: US grade 6).^14-16^ A US grade level of 6 is the equivalent to a UK year level of 7 (11–12 years). As the score increases, the readability of the website decreases.

The FKGL, SMOG Index and GFI are calculated using the following formulas: 0.39×(total number of words ÷ total number of sentences)+11.8×(total number of syllables ÷ total number of words) − 15.59; 1.0430 (√ total number of polysyllabic words×(30 ÷ total number of sentences)+3.1291; and 0.4×[(total number of words ÷ total number of sentences)+100×(total number of complex words ÷ total number of words)].

In the UK, the Government recommends a reading age of 9 years for online information.^17^ The NHS Digital service manual team advises that medical pages in the NHS should aim for a reading age of 9–11 years.^18^ Therefore, a reading age of 9–11 years was used as the standard for this study. This is the equivalent to the US grades of 4–6.

### Quality assessment

The quality of the online information was assessed using the DISCERN instrument. This is a standardised 16-item checklist, rated on a 5-point scale, that has been organised into three parts: (a) publication reliability (questions 1–8), (b) treatment information quality (questions 9–15) and (c) overall publication quality (question 16).^19^ The score for question 16 reflects the overall quality of the website information. An average score of 1 (low/poor quality) represents serious or extensive shortcomings, whereas a score of 5 (high/good quality) reflects minimal or no shortcomings.

### Subgroup analyses

Subgroup analyses were performed to overcome any effects of variation in the search terms used and the type of media assessed on the readability and quality of the online information. The websites in the main dataset were divided into two subgroups: group one for medical, physician and academic websites; and group two for media, social media and discussion websites. The websites were also divided into search-term-specific subgroups.

### Statistical analysis

Statistical analyses were performed using the data analysis tool on Microsoft Excel 2019 statistical software. The mean and 95% confidence interval (CI) for the readability and quality scores were generated for websites in the main dataset, as well as for websites in each subgroup. Readability and quality scores were compared between subgroups using two-tailed unpaired statistical *t*-tests to gauge statistical significance. The Spearman’s rank-order correlation coefficient was calculated to measure the strength and direction of association between the FRES and DISCERN instrument scores of websites in the main dataset. Statistical significance was set at *p*<0.05.

## Results

Of the 100 websites generated by the ‘anosmia’ and ‘loss of smell’ searches, 79 met the eligibility criteria (Figure 1). Reasons for exclusion of 21 websites were as follows: website duplication (*n*=16), inaccessibility (*n*=1), nonwritten information (*n*=1) and irrelevant information (*n*=3). Websites providing only information regarding the definition and etymology of anosmia, contact details for a clinic or access to a database search results page were classified as being ‘irrelevant’. Furthermore, 80% (*n*=63) of websites in the main dataset were identified as being group one sources, whereas 20% (*n*=16) were identified as being group two sources (Table 1).

**Table 1:** Summary of readability and quality between group one (medical, physician and academic media) and group two (media) websites

Test name	Group one (*n*=63)	Group two (*n*=16)	*p* value*
FRES, mean (95% CI)	44.54 (40.52–48.57)	53.27 (49.27–57.27)	0.0374
FKGL, mean (95% CI)	12.41 (11.55–13.28)	10.37 (9.57–11.17)	0.0236
SMOG Index, mean (95% CI)	11.03 (10.39–11.66)	9.39 (8.79–10.00)	0.0141
GFI, mean (95% CI)	15.13 (14.22–16.03)	12.62 (11.72–13.52)	0.00820
DISCERN score, mean (95% CI)	2.98 (2.73–3.23)	2.58 (2.21–2.95)	0.132

*p<0.05 for significance.

CI = confidence interval; FKGL = Flesch–Kincaid Grade Level; FRES = Flesch Reading Ease Score; GFI = Gunning Fog Index; SMOG = Simple Measure of Gobbledygook

### Readability assessment

The mean and 95% CI for the FRES, FKGL, SMOG and GFI scores of websites in the main dataset were 46.31 (42.94–49.68), 12.00 (11.27–12.73), 10.69 (10.16–11.23) and 14.62 (13.85–15.39), respectively (Table 2). The mean FRES equates to ‘difficult’ readability. The mean FKGL, SMOG Index and GFI scores equate to reading ages of 17–18 years, 16–17 years and >17 years, respectively. The websites in the main dataset with a reading age of 9–11 years were authored by Healthwise,^20^ a nonprofit company that produces health content (FKGL and SMOG scores of 4.7 and 5.3 (reading age of 10–11 years), respectively), the NHS (SMOG score of 5.6, reading age of 11–12 years)^21^ and the Mayo Clinic^22^ (SMOG score of 6.3, reading age of 11–12 years).

**Table 2:** Summary of readability and quality data for ‘anosmia’ and ‘loss of smell’ search terms

Test name	Main dataset search term analysis	Search-term-specific subgroup analysis		
	‘Anosmia’ and ‘Loss of smell’ (*n*=79)	‘Anosmia’ (*n*=46)	‘Loss of smell’ (*n*=46)	*p* value*
FRES, mean (95% CI)	46.31 (42.94–49.68)	41.40 (36.56–46.23)	50.80 (47.11–54.49)	0.00248
FKGL, mean (95% CI)	12.00 (11.27–12.73)	12.78 (11.70–13.85)	11.39 (10.56–12.22)	0.0428
SMOG Index, mean (95% CI)	10.69 (10.16–11.23)	11.35 (10.56–12.14)	10.24 (9.62–10.86)	0.0275
GFI, mean (95% CI)	14.62 (13.85–15.39)	15.55 (14.45–16.65)	13.94 (13.07–14.82)	0.0239
DISCERN score, mean (95% CI)	2.90 (2.69–3.11)	3.29 (3.00–3.57)	2.50 (2.30–2.69)	0.0000148

*p<0.05 for significance

CI = confidence interval; FKGL = Flesch–Kincaid Grade Level; FRES = Flesch Reading Ease Score; GFI = Gunning Fog Index; SMOG = Simple Measure of Gobbledygook

#### Subgroup analysis: type of media

The mean and 95% CI for the FRES, FKGL, SMOG Index and GFI scores of group one websites were 44.54 (40.52–48.57), 12.41 (11.55–13.28), 11.03 (10.39–11.66) and 15.13 (14.22–16.03), respectively (Table 1). The mean FRES equates to ‘difficult’ readability. The mean FKGL, SMOG Index and GFI scores were the equivalent to reading ages of >17 years, 16–17 years and >17 years, respectively.

The mean and 95% CI for the FRES, FKGL, SMOG Index and GFI scores of group two websites were 53.27 (49.27–57.27), 10.37 (9.57–11.17), 9.39 (8.79–10.00) and 12.62 (11.72–13.52), respectively (Table 1). The mean FRES equates to ‘fairly difficult’ readability. The mean FKGL, SMOG Index and GFI scores equate to reading ages of 15–16 years, 14–15 years and >17 years, respectively. In comparison between group two websites, group one websites had significantly lower readability (Table 1).

#### Subgroup analysis: search terms

The mean and 95% CI for the FRES, FKGL, SMOG Index and GFI scores of websites generated by the use of the ‘anosmia’ search term were 41.40 (36.56–46.23), 12.78 (11.70–13.85), 11.35 (10.56–12.14) and 15.55 (14.45–16.65), respectively (Table 2). The mean FRES equates to ‘difficult’ readability. The mean FKGL, SMOG Index and GFI scores equate to reading ages of >17 years, 16–17 years and >17 years, respectively.

The mean and 95% CI for the FRES, FKGL, SMOG Index and GFI scores of websites generated by the use of the ‘loss of smell’ search term were 50.80 (47.11–54.49), 11.39 (10.56–12.22), 10.24 (9.62–10.86) and 13.94 (13.07–14.82), respectively (Table 2). The mean FRES equates to ‘fairly difficult’ readability. The mean FKGL, SMOG Index and GFI scores equate to reading ages of 16–17 years, 15–16 years and >17 years, respectively. In comparison with the use of the ‘anosmia’ search term, websites generated by the use of the ‘loss of smell’ search term were significantly more readable (Table 2).

### Quality assessment

The mean and 95% CI for the DISCERN instrument scores of websites included in the main dataset were 2.90 (2.69–3.11) (Table 2). This equates to ‘moderate’ information quality. The websites in the main dataset with the highest quality were peer-reviewed academic articles by Burges Watson *et al*,^23^ Hornuss *et al*^24^ and Butowt and Von Bartheld.^25^ Their DISCERN instrument scores were 4.80, 4.70 and 4.64, respectively, which all equate to ‘high’ quality.

#### Subgroup analysis: type of media

There was no significant difference in the quality of information between group one and group two websites (Table 1). The mean and 95% CI for the DISCERN instrument scores for group one and two websites were 2.98 (2.73–3.23) and 2.58 (2.21–2.95), respectively (*p*=0.132). These scores equate to ‘moderate’ quality.

#### Subgroup analysis: search terms

In comparison with the use of the ‘loss of smell’ search term, the quality of online information generated by the use of the ‘anosmia’ search term was significantly higher (Table 2). The mean and 95% CI for the DISCERN instrument scores for websites generated by the ‘anosmia’ and ‘loss of smell’ searches were 3.29 (3.00–3.57), and 2.50 (2.30–2.69), respectively (*p*=0.0000148). For both groups, the mean DISCERN instrument scores equate to ‘moderate’ quality.

### Readability and quality association

**Figure 2:**
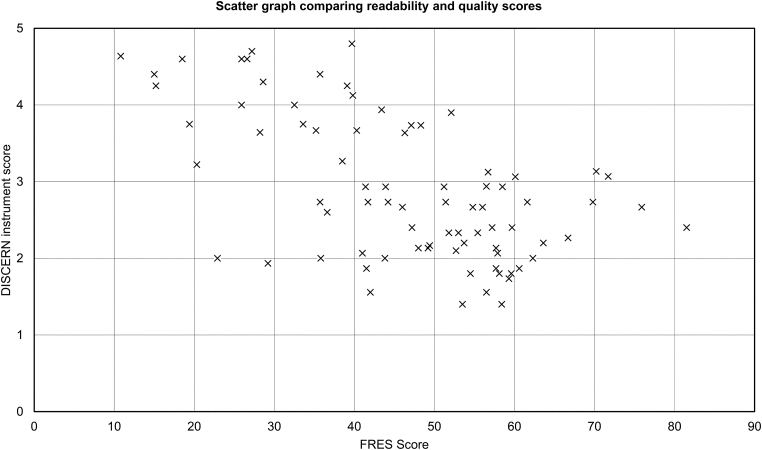
Scatter graph of FRES against DISCERN score. Significant negative correlation was detected (*r*_s_=−0.500; *p*=0.0000148). FRES = Flesch Reading Ease Score

A statistically significant negative correlation was found between information readability (FRES) and quality (DISCERN instrument) scores for websites in the main dataset (*r*_s_=−0.500; *p*<0.05; Figure 2).

## Discussion

Anosmia is a debilitating symptom that can have a marked impact on overall wellbeing and quality of life. Patients with the inability to smell commonly experience psychosocial issues, including anhedonia and generalised anxiety, that may be associated with increased morbidity and mortality.^26^ More immediate implications of living with olfactory disturbances include the inability to detect environmental hazards such as gas leaks or fires. This is significant as, due to an ageing population and COVID-19, the prevalence of anosmia will continue to rise, with increasing numbers of patients seeking online health-related information before medical review. This is the first study, to our knowledge, to have systematically evaluated the readability and quality of online information on anosmia.

The readability of online information on ‘anosmia’ and ‘loss of smell’ was largely inadequate. The unique websites in the main dataset had a mean reading age of 16+ years, which far exceeds the UK government and NHS standard of 9–11 years for online information.^17,18^ Notably, subgroup analyses demonstrated that the use of the lay search term ‘loss of smell’ generated websites with significantly higher readability. Despite this, even the lowest mean reading age generated (15–16 years) was above the recommended standard.

Furthermore, group two websites were found to be significantly more readable than group one websites. As with the search term-specific subgroup analysis, even the lowest mean reading age generated (14–15 years) was above the recommended standard. Thus, encouraging the use of simpler search terms is not sufficient to identify websites with appropriate readability. These findings are concerning as online information of low readability can be difficult for patients to understand and interpret, particularly for those from a lower socioeconomic status where limited health literacy is prevalent. As a result, the information may be used inappropriately and could result in worse health outcomes.^27^

In terms of quality, the mean DISCERN instrument score for websites in the main dataset equates to ‘moderate’ quality, representing potentially important but not serious shortcomings. Notably, this classification was unaffected by the type of media analysed and no significant difference in DISCERN instrument scores was found between subgroups. Nevertheless, group one websites produced a higher mean DISCERN instrument score. This finding is unsurprising as medical, physician and academic websites are more likely to have undergone extensive peer-review before publication than social media and discussion websites. Furthermore, the mean DISCERN instrument score was significantly higher when ‘anosmia’ was used as the search term. This demonstrates that encouraging the use of medical search terms and the identification of group one websites may direct patients to online information of higher quality.

Our readability and quality findings are in line with existing literature in otolaryngology. With a potential to affect the informed consent process, Grose *et al* found online information on nasal septoplasty to be of poor readability and quality.^28,29^ Similarly, Ting and Hu found that thyroplasty-related online information is of suboptimal readability and quality.^30^ These findings suggest that greater work is required in the field to support patient decision-making and to achieve improved health outcomes.

Notably, websites in the main dataset with readability scores equating to the target reading age of 9–11 years were authored by a nonprofit company that develops health content (Healthwise), a hospital (the Mayo Clinic) and the NHS. Websites of the highest quality were academic websites.

When the readability and quality scores were correlated, a negative correlation between the FRES and DISCERN instrument scores emerged. This can be interpreted as online information becoming more readable as its quality decreased. Ensuring that online information on anosmia is both easy to read and of high quality can prove challenging. Our findings demonstrate the need to not only educate patients to perform improved searches, but also to revise online information on anosmia to achieve these quality standards.

We recommend online information be produced by qualified clinicians while adopting NHS Digital guidelines and incorporating patient and public involvement. Ideally, regular expert review and revision of online information should be undertaken to ensure information is compatible with the latest evidence base. In the meantime, given the paucity of high-quality online information on anosmia that is easy to read, healthcare providers should familiarise themselves with readable and accurate online resources and direct patients accordingly.

We recommend that patients are directed to websites produced by health providers such as the NHS and hospitals, or nonprofit organisations that develop material for patient health education, to enable them to access readable information. For the most accurate information, patients could be directed to peer-reviewed academic websites. However, these websites may have low readability. Thus, the educational background of the patient should be taken into consideration.

### Limitations

Our study has some limitations. Firstly, the Internet is a dynamic platform whereby online information is constantly being updated. Therefore, the websites assessed in this study may not necessarily reflect the information accessible to patients at another point in time. Secondly, Google was the only search engine used to identify anosmia-related online information. As a result, the findings discussed may not truly be representative of the patient experience. Moreover, other jurisdictions that predominantly use different search engines would likely reveal different information sources. Additionally, non-English websites were excluded from our study. To accommodate a diverse and multilingual patient population, it is important to consider online material in different languages. Finally, readability evaluation tools fail to analyse audio, image, or video-based information. These are key communication tools that add clarity and comprehensibility to online material.

## Conclusion

Anosmia is a significant and recognised symptom of COVID-19 that can prompt patients to seek urgent health-related information on the Internet. The current online information on anosmia is of moderate quality and written above the recommended reading age guidance in the UK. Healthcare professionals including ENT surgeons have a responsibility to advocate for improved online patient information on anosmia and direct patients towards high-quality resources that are written for the layperson. This will improve patient understanding and aid the shared decision-making process.

## Authorship statement

HR conceived the idea of writing the manuscript. HR and SL designed the methodology, acquired and analysed the data, drafted and revised the manuscript. HR and SL approved the manuscript for submission. HR agrees to be accountable for all aspects of the work
